# Early and late mortality in elderly patients after hip fracture: a cohort study using administrative health databases in the Lazio region, Italy

**DOI:** 10.1186/1471-2318-11-37

**Published:** 2011-08-05

**Authors:** Esmeralda Castronuovo, Patrizio Pezzotti, Antonella Franzo, Domenico Di Lallo, Gabriella Guasticchi

**Affiliations:** 1 Lazio Sanità - Agenzia di Sanità Pubblica, Rome, Italy; 2 Direzione Centrale Salute - Regione Autonoma Friuli Venezia Giulia, Italy

## Abstract

**Background:**

Hip fractures represent one of the most important causes of morbidity and mortality in elderly people. We evaluated the risk and the potential determinants of early, mid and long term mortality, in a population-based cohort of subjects aged ≥ 65 years old.

**Methods:**

Using hospital discharge database we identified all hospitalized hip fracture cases of 2006, among residents in Lazio Region aged ≥ 65 years old. The mortality follow-up was performed through a deterministic record-linkage between the cohort and the death registry for the years 2006 and 2007.

Kaplan-Meier method was used to calculate cumulative survival probability after admission. Shared frailties Cox regression model was used to estimate adjusted hazard ratios (HRs) for early (within 1 month), mid (1-6 months) and long term (6-24 months) mortality. As possible cofactors we considered age, gender, marital status, education degree, comorbidities, surgical intervention, and hospital volume of surgical treatment for hip fracture.

**Results:**

We identified 6,896 patients; 78% were females, median age was 83 and 9% had two or more comorbidities. Five percent died during hospital stay; the cumulative probability of dying at 30, 180 days, and at 2 years was 7%, 18% and 30%. In the first month following admission, we found a significantly increased HR with older age, male sex, not married status, history of hearth disease, chronic pulmonary and renal disease; for those who had surgery there was a significantly increased HR within two days after surgical intervention and a significantly decreased HR thereafter compared to those who received a conservative management. Between 1 and 6 months significantly increased HRs were for older age, male sex and higher hospital volume of surgical treatment. After six months, significantly increased HRs were for older age, male sex, presence of dementia and other low prevalence diseases.

**Conclusion:**

In Lazio region the risk of dying after hip fracture is similar to that found in high-income countries. Both clinical and organizational factors of acute care are associated with the risk of early mortality. As time passes, some of these factors tend to become less important while older age, male gender, the presence of cognitive problems and the presence of other comorbidities remain significant.

## Background

Hip fracture is a common injury in the elderly associated with significant morbidity, mortality and disability. Incidence increases with age, and 75% of hip fractures occur in women [1,2]. About 50% of patients who lived independently before sustaining a hip fracture are unable to regain their independent lifestyle [3-5]. Five to 12% of hip fracture patients discharged to a post-acute care facility were readmitted to the hospital within 6 weeks [6]. The social and economic costs on patients, their families, and society at large are vast. In 2002, more than 86,000 hip fractures were recorded in Italy in patients over 45 years of age; persons over 65 years accounted for 93% of those hospital admissions and 73% of these were females. The direct cost of hospitalisation in Italy, in patients over 65, was almost 400 million euros [7].

Mortality associated with hip fractures has been estimated about 5-10% within one month, and around 20%-30% of patients die within one year [4,8,9]. A review of the outcomes after hip fracture over a forty-year period (1959-1998) reported that mortality at 6 and 12 months afterwards remained essentially unchanged over the period reviewed [10]. Males have a higher risk of mortality and lose more years of life proportionally; this higher risk has been shown to persist for up to 10 years [11]. This is also confirmed by an Italian study on 493 cases of proximal femur fractures in patients over 65 years of age that estimated the probability of death one year after hip fracture at 20.8% in women, and 30.9% in men [12].

Surgical treatment within 24-48 hours after hip fracture is recommended by clinical guidelines[13-15] but the effect of this suggestion on patient morbidity and mortality is controversial. Some studies have reported no differences in outcomes between delayed and immediate treatment [16,17], others reported that for patients without comorbidities, mortality increases only if surgery is delayed beyond the fourth day [9].

Recent studies report that the combination of this trauma with a co-morbidity represents a large percentage of mortality [1,6,18,19]. A history of congestive heart failure, chronic obstructive pulmonary disease, dementia, cancer, and malignancy increase the risk of mortality after hip fracture [20].

Although the various factors associated with increased risk of mortality after hip fracture are well-recognized, there have been very few rigorously developed and executed multivariable risk models capable of evaluating the roles of the determinants for either short term or long term fatal outcome [18]. Accordingly, we had two main objectives in this study: (1) to estimate, in a population-based cohort of subjects aged ≥ 65 years old, early (within 1 month after admission), mid term (from one to six months) and long term (from 6 to 24 months) mortality after the hospital admission immediately following the hip fracture, (2) to evaluate potential determinants of early, mid and long term mortality.

## Methods

### Context of the study

The Lazio region (central Italy) is the third most densely populated region in Italy and has a resident population of about 5,600,000 people (19.7% are aged 65 or more). Each year, hip fractures are the most common injury leading to hospital admission among the elderly, with an incidence between 1.3 per 1,000 for subjects aged 65-69 years old, and 34.3 for those aged 90 years old or more.

### Study Population

We conducted a prospective cohort study of elderly hospitalised hip fracture patients.

Data were extracted from Regional Hospital Discharge (RHD) database that reports specific information from all discharges from hospitals in the region. The details have been described elsewhere [21]. Briefly, RHD was started in 1994 and all hospitals in the region are required to use a standardized form that records admission and discharge dates, personal data of the patient (i.e., date of birth, gender, name, surname, municipality of residence, nationality), the principal diagnosis and up to five secondary diagnoses [coded by the International Classification of Diseases - ninth revision (ICD-9)], surgical, therapeutic and diagnostic procedures (also coded by the ICD-9), and death, if it occurred during the hospital admission. We selected patients admitted to an acute care hospital between 1^st ^January and 31^st ^December 2006, with a main diagnosis of hip fracture, aged 65 or older who were residents of the Lazio region. Hip fracture patients were identified according to ICD-9 coding: fractures of the neck of the femur (ICD-9 820.xx) and fracture of other and unspecified parts of the femur (ICD-9 821.xx). Hip fracture patients who had, in the 24 months prior to the index admission, a previous hip fracture or had a malignant neoplasm diagnosis (diagnoses codes 140.xx-239.xx), were excluded from the analysis. Patients who suffered trauma to a site other than the lower extremities (codes Diagnosis Related Group: 484-487) were also excluded.

To follow-up on mortality, we developed a multi-stage, deterministic record-linkage between the cohort and the regional mortality registry for the years 2006-2007 using two match keys: first by full name, gender, date of birth and municipality of birth (step 1) and secondly by tax identification number (step 2). A visual check was then performed for cases that matched only with one of the two criteria (step 3); in this case the matching was not accepted if there was an inconsistency (e.g., the date of death was preceding the date of hospital admission). Similarly, we performed a record linkage of the cohort with the RHD, for the years 2006-2007, in order to identify hospital readmissions after the hospital admission following the hip fracture. We analogously linked the cohort data with the Inpatient Rehabilitation Facilities databases [22,23] to evaluate access to post-acute rehabilitation care after discharge from acute care.

### Statistical Analysis

The outcome studied was the time to death after hospital admission for hip fracture. We used survival analysis techniques such as Kaplan-Meier curves to calculate cumulative survival probability after hospital admission and up to 2 years later and Cox regression model. We assumed that patients not listed in the regional mortality registry were alive, as of December, 31, 2007.

We decided to perform, after a preliminary analysis, survival analyses stratified by follow-up period [i.e., within 30 days (early), from 30 to 180 days (mid-term), and more than 180 days (long-term)]. Specifically, we performed separate analyses to evaluate survival at 30 days, between 30 and 180 days, and from 180 days up to the end of follow-up (maximum 2 years). There are two reasons for this approach: 1) to identify factors associated with mortality after hip fracture whose effect changed as time elapses; 2) to guarantee the proportionality requests assumption of the Cox model.

We applied a shared frailties Cox regression model to take into account that patients were clustered within the hospital where they were recovered. Shared frailty Cox models provide a useful extension of standard survival models by introducing a random effect (frailty) when the survival data are correlated [24].

The assumption of proportional hazards was checked, using graphical techniques and a detailed goodness-of-fit test for each parameter based on the scaled Schoenfeld residuals obtained from the Cox regression model.

### Covariate definitions

The factors evaluated were those available in the RHD database: age (divided into three groups: 65-74; 75-84; 85+), gender, marital status [married, not married (it includes not married, widowed and divorced)], years of education (≤ 8 and > 8 years of school), type of fracture (neck of femur, other and unspecified parts of femur), presence of co-morbid conditions (see below for details), hospital annual hip fracture surgery volume (i.e., ≤ 45, 46-200; > 200 surgeries per year), and elapsed time to surgery. This last factor was analyzed as a time dependent variable and the effect of surgery was separately evaluated to distinguish its effect whether the surgery was done within the first two days from that thereafter. More exactly, for each patient this variable was initially set to no surgery; then, for those who had surgical intervention, the variable was set to surgery from the day of surgery. We thus created 5 categories: no surgery, surgery within two days of admission and elapsed time after surgery ≤ 2 days, surgery within two days of admission and elapsed time after surgery > 2 days, surgery after two days of admission and elapsed time after surgery ≤ 2 days, surgery after two days since admission and elapsed time after surgery > 2 days.

Comorbidities were identified according to the modified Charlson index - the Deyo index [25,26], using all hospital discharges in the years 2004-2005. We defined 16 categories (i.e., myocardial infarction, congestive heart failure, peripheral vascular disease, cerebrovascular disease, dementia, chronic pulmonary disease, rheumatologic disease, peptic ulcer disease, mild liver disease, moderate to severe liver disease, diabetes, diabetes with chronic complications, hemiplegia or paraplegia, renal disease, any malignancy including leukaemia and lymphoma, and acquired immunodeficiency syndrome). One comorbid condition that was in the Deyo list was not considered because we had excluded from the cohort all subjects with a diagnosis of cancer. In survival analyses comorbidities with a frequency of < 4% were grouped as "other comorbidities". Finally, to evaluate the effect of entering in in-patient rehabilitation facilities we used a dummy time dependent variable that was set to 0 before rehabilitation facility admission and one thereafter.

Data were analyzed using Stata software (Stata Corporation, Version 11).

### Study approval

Lazio Sanità - Agenzia di Sanità Pubblica is the governmental agency of the Lazio region responsible for health information systems (e.g., infectious disease notifications, hospital discharge records); the management of these data for public health purposes does not require a patient's informed consent. Data management is performed in respect with the requirements of the current privacy laws in Italy.

The authors declare that they had permission to access and use the databases from which they extracted the data for the specific analyses

## Results

### Characteristics of the cohort

Initially, we identified 7,845 subjects aged 65 year old or more (74.7% of the total hospital admissions for hip fracture in 2006 in the region). After exclusions (269 cases not residents of the Lazio region, 81 who suffered major trauma to a site other than the lower extremities, 231 who had at least one previous hip fracture recorded in the 24 months prior to the index admission, and 368 who had a previous malignant neoplasm diagnosis recorded in the 24 months prior to the index admission) the study population was composed of 6,896 subjects.

Of 6,896 patients, 78% were female. The age of the patients ranged from 65 to 106 years, with a median of 83 [interquartile range (IQR) 78-88]: for women and 82 (IQR:76-86) for men. Forty-eight percent were not married and this condition was more prevalent among women (53.3%). More than 50% had a low educational level. The majority of patients had a femur neck fracture (92.5%), 1,117 patients (16.1%) received conservative treatment without surgery and 56.8% were admitted to a hospital with medium surgical volume for hip fracture. After hospital discharge, 50% of the patients received rehabilitation care and 20.1% were readmitted to the hospital (table 1).

**Table 1:** Patient general characteristics and deaths at 30, 180 and after 180 days mortality from admission (N = 6.986). Lazio Region, Italy, 2006-2007

				DEATHS %			
**Variables**	**Characteristics**	**Patients** **N %**		**30 days (N = 437)**	**30-180 days (N = 777)**	**> 180 days (N = 581)**	**Total** **(N = 1,795)**

**Age**	65-74	1,067	15.5	3.3	3.5	4.4	11.2
	75-84	3,103	45.0	4.2	9.0	7.5	20.8
	85+	2,726	39.5	9.8	16.9	11.0	37.8
**Gender**	Female	5,387	78.1	5.0	10.0	7.9	23.0
	Males	1,509	21.9	11.1	15.6	10.1	36.9
**Marital Status**	Married	3,574	51.8	5.0	10.4	8.1	23.6
	Not married	3,322	48.2	7.6	12.2	8.8	28.6
**Years of education**	≤ 8 years	855	12.4	5.2	7.5	7.7	20.5
	> 8 years	6,041	87.6	6.5	11.8	8.5	26.8
**Type of fracture**	Neck of femur	6,378	92.5	6.3	11.3	8.5	26.2
	Other parts of femur	518	7.5	6.6	10.8	7.1	24.5
**Surgical delay**	Conservative treatment	1,117	16.2	16.2	15.9	10.3	42.5
	0-2 days	808	11.7	4.6	8.4	9.8	22.8
	2 days or more	4,971	72.1	4.4	7.1	7.8	19.2
**Hospital annual hip fracture surgeries volume**	≤ 45	548	7.9	5.1	6.8	9.1	21.0
	46-200	3,914	56.8	6.4	12.3	8.5	27.2
	> 200	2,434	35.3	6.5	10.6	8.2	25.3
**Rehabilitation care access**	yes	3,404	49.4	0.9	9.6	8.2	18.7
	no	3,492	50.6	11.7	12.9	8.6	33.2
**Hospital readmissions**	yes	1,388	20.1	1.2	23.3	14.6	39.0
	no	5,508	79.9	7.6	8.2	6.9	22.7
**Total**		6,896	100.0	6.3	11.3	8.4	26.0

% of in-hospital-mortality during first hospital admission out of the total deaths				65.9	7.4	0.0	19.2

Seventy percent (4,850) had no identified comorbidity; 20.6% had one, 6.1% had two, and 3% had three or more comorbidities. Table 2 lists the comorbidities recorded and their frequencies. The most common were diabetes (10.6%), chronic pulmonary disease (9.1%), cerebrovascular disease (6.3%) and cardiovascular disease (4.2%).

**Table 2:** Comorbidities identified in the previous two years since hospital admission for hip fracture according to the modified Charlson index by Deyo.

Diagnostic category	ICD9-CM codes	n. subjects with disease (N = 2,046)	
Diabetes	250-250.3*; 250.7	741	10.6
Chronic pulmonary disease	490-496*; 500-505*; 506.4*	634	9.1
Cerebrovascular disease	430;438*	438	6.3
Heart failure	428-428.9	294	4.2
Renal disease	582-582.9*; 583-583.7*; 585*; 586*; 588-588.9*	265	3.8
Dementia	290-290.9*	236	3.4
Mild liver disease	571.2*; 571.5*; 571.6*; 571.4-571.49	79	1.1
Myocardial infarction	410-410.9; 412*; 785.4*; v43.4*; Procedure 38.48	56	0.8
Hemiplagia or paraplegia	344.1*; 342.9*	50	0.7
Peripheral vascular disease	443.9*;441-441.9*	38	0.5
Dibetes with chronic complication	250.4-250.6*	33	0.5
Disease of connettive tissue	710*;710.1*; 710.4*; 714-714.2*; 714.81*; 725*	33	0.5
Peptic ulcer disease	531-534.9; 531.4-531.7; 532.4-532.7; 533.4-533.7; 534.4-534.7	23	0.3
Moderate or severe liver disease	572.2-572.8*	12	0.2
AIDS	042-044	1	0.0

Lazio Region, Italy, 2006-2007

*Asterisc codes were included if listed during index or prior admission. Other codes were included only if recorded prior to the index admission

During a median follow-up time of 15.8 months (range 1 day-2 years) there were 1,795 deaths, corresponding to 26% of the enrolled patients (23% females, 37% males). Table 1 shows the percentage of deaths by time since admission and by the main characteristics of the patients. About 5% of patients (346) died during the hospital admission following the hip fracture [288 in the first month (16%) and 58 at six months (3.2%)], 6.3% died within one month after admission, 11.3% after one month within six months and 8.4% within two years.

**Figure 1:**
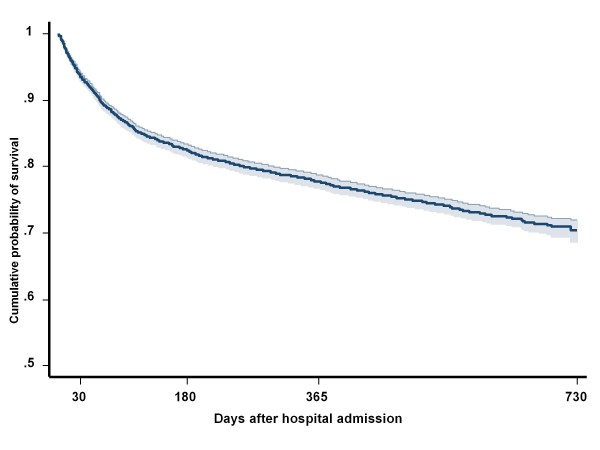
**Two year Kaplan-Meier estimates (with 95% CI) of the cumulative probability of survival after hospital admission for hip fracture**.

Figure 1 shows Kaplan-Meier estimates of the cumulative probability of survival after hospital admission for hip fracture. The probability of survival at one month, six months and at two years were 0.93 (95%CI: 0.93, 0.94) 0.82 (95%CI: 0.81, 0.83), 0.70 (95%CI: 0.68, 071), respectively. The risk of death dramatically changed during the study period from being extremely high in the first month [0.79 per person-year (PY) 95%CI: 0.72, 0.87], and then declining thereafter (0.31, 95%CI: 0.29, 0.33) and 0,10 (95%CI: 0.99, 0.11) per PY between 1 and six months and after six months, respectively).

Tables 3, 4 and 5 show the adjusted hazard ratios (HRs) and 95% CIs estimated by the shared frailties Cox regression models referring to the three time-periods considered.

**Table 3:** Shared frailties Cox regression model: Hazard Ratio (HR) of mortality in the period 1-30 days after hospital admission.

Variables	Characteristics	Adjusted HR*	95%CI	
**Age (reference group: 65-74)**	75-84	1.19	0.82	1.72
	85+	2.58	1.80	3.69
**Gender (vs. Females)**	Males	2.50	2.04	3.06
**Marital Status (vs. Married)**	Not married	1.56	1.26	1.91
**Years of education (vs. ≤ 8 years)**	> 8 years	0.94	0.67	1.30
**Type of fracture (vs. Neck of femur)**	Fracture of other and unspecified parts of femur	1.05	0.73	1.50
**Elapsed time from admission to surgery and time after surgery (vs. No surgery)**	Surgery within 2 days and time after surgery ≤ 2 days	1.82	0.77	4.28
	Surgery within 2 days and time after surgery > 2 days	0.54	0.35	0.83
	Surgery after 2 days and time after surgery ≤ 2 days	1.45	1.01	2.07
	Surgery after 2 days and time after surgery > 2 days	0.64	0.50	0.82
**Hospital annual surgery for hip fracture volume [vs. Low (≤ 45)]**	Medium (46-200)	1.12	0.72	1.75
	High (> 200)	1.19	0.75	1.90
**Rehabilitation care access (vs. No)**	Yes	0.39	0.26	0.58
**Comorbid condition (vs. No comorbidity)**	Heart failure	2.59	1.93	3.49
	Cerebrovascular disease	1.06	0.75	1.49
	Chronic pulmonary disease	1.48	1.13	1.94
	Diabetes	0.89	0.65	1.21
	Renal disease	1.92	1.38	2.67
	Dementia	1.12	0.71	1.77
	Other	1.36	0.93	1.99

Lazio region, Italy, 2006-2007

*Theta < 0.05: p-value of LR test comparing Cox frailty model to standard Cox

**Table 4:** Shared frailties Cox regression model: Hazard Ratio (HR) of mortality in the period 30-180 days after hospital admission.

Variables	Characteristics	Adjusted HR*	95%CI	
**Age (vs. 65-74)**	75-84	2.73	1.93	3.85
	85+	5.67	4.03	7.99
**Gender (vs. Females)**	Males	1.88	1.59	2.20
**Marital Status (vs. Married)**	Not married	1.08	0.92	1.26
**Years of education (vs. ≤ 8 years)**	> 8 years	0.75	0.57	0.99
**Type of fracture (vs. Neck of femur)**	Fracture of other and unspecified parts of femur	1.08	0.82	1.43
**Elapsed time from admission to surgery and time after surgery (vs. No surgery)**	Surgery within 2 days and time after surgery ≤ 2 days	NE	-	-
	Surgery within 2 days and time after surgery > 2 days	0.54	0.41	0.73
	Surgery after 2 days and time after surgery ≤ 2 days	2.26	0.31	16.33
	Surgery after 2 days and time after surgery > 2 days	0.63	0.52	0.76
**Hospital annual surgery for hip fracture volume [vs. Low (≤ 45)]**	Medium (46-200)	1.69	1.17	2.46
	High (> 200)	1.52	1.02	2.27
**Rehabilitation care access (vs. No)**	Yes	0.85	0.73	0.99
**Comorbid condition (vs. No comorbidity)**	Heart failure	1.74	1.31	2.31
	Cerebrovascular disease	0.96	0.73	1.26
	Chronic pulmonary disease	1.2	0.95	1.51
	Diabetes	1.34	1.08	1.66
	Renal disease	1.27	0.92	1.76
	Dementia	2.18	1.65	2.88
	Other	1.50	1.11	2.03

Lazio Region, Italy, 2006-2007

*Theta < 0.05: p-value of LR test comparing Cox frailty model to standard Cox; NE: not estimable

**Table 5:** Shared frailties Cox regression model: Hazard Ratio (HR) of mortality up180 days after hospital admission.

Variables	Characteristics	Adjusted HR*	95%CI	
**Age (vs. 65-74)**	75-84	1.94	1.41	2.66
	85+	3.51	2.56	4.83
**Gender (vs. Females)**	Males	1.57	1.29	1.90
**Marital Status (vs. Married)**	Not married	0.99	0.83	1.18
**Years of education (vs. ≤ **8 **years)**	> 8 years	0.89	0.67	1.17
**Type of fracture (vs. Neck of femur)**	Fracture of other and unspecified parts of femur	0.83	0.59	1.17
**Elapsed time from admission to surgery and time after surgery (vs. No surgery)**	Surgery within 2 days and time after surgery ≤ 2 days	NE	-	-
	Surgery within 2 days and time after surgery > 2 days	0.80	0.59	1.08
	Surgery after 2 days and time after surgery ≤ 2 days	NE	-	-
	Surgery after 2 days and time after surgery > 2 days	0.62	0.49	0.77
**Hospital annual surgery for hip fracture volume [vs. Low (≤ 45)]**	Medium (46-200)	1.06	0.75	1.49
	High (> 200)	1.04	0.71	1.51
**Rehabilitation care access (vs. No)**	Yes	0.93	0.78	1.10
**Comorbid condition (vs. No comorbidity)**	Heart failure	1.41	0.97	2.07
	Cerebrovascular disease	1.07	0.78	1.47
	Chronic pulmonary disease	1.19	0.90	1.58
	Diabetes	1.16	0.90	1.50
	Renal disease	1.41	0.97	2.07
	Dementia	1.85	1.28	2.69
	Other	1.69	1.19	2.40

Lazio Region, Italy, 2006-2007

*Theta < 0.05: p-value of LR test comparing Cox frailty model to standard Cox; NE: not estimable

Eight statistically significant factors increased the risk of death within 30 days of a hip fracture: age ≥ 85 years old (HR: 2.58, 95%CI: 1.80-3.69 compared to 65-74 years old), male gender (HR:2.50, 95%CI: 2.03-3.05), unmarried status (HR: 1.55, 95%CI: 1.26-1.91), surgery after two days since admission and elapsed time after surgery ≤ 2 days (HR: 1.45, 95%CI: 1.01-2.07), history of heart disease (HR: 2.59, 95%CI: 1.93-3.49); chronic pulmonary disease (HR:1.48, 95%CI:1.13-1.94), and renal disease (HR:1.92, 95%CI:1.38-2.67). There was a significant risk reduction for those who accessed rehabilitative care (HR:0.39, 95%CI: 0.26,0.58) and surgery (both within two days and after two days of admission) and elapsed time after surgery > 2 days (HR:0.54, 95%CI: 0.35-0.83 and HR:0.64, 95%CI: 0.50-0.82 for those who had a surgery within and after two days, respectively).

HR of time to death in the period one-to-six-months after hospital admission were significantly higher for people aged 75-84 and ≥ 85 years (HR: 2.73, 95%CI: 1.93-3.85; HR: 5.67, 95%CI 4.03-7.99, respectively), male gender (HR: 1.88, 95%CI: 1.59-2.20) and for hospitals with higher surgical volume (HR: 1.69, 95%CI 1.17-2.46). There was a significant risk reduction for those accessing rehabilitative care (HR:0.85, 95%CI: 0.73-0.99) and for people who had a surgery intervention (after two days since surgery) (HR:0.54, 95%CI: 0.41-0.73; HR:0.63, 95%CI: 0.52-0.76 within and after two days from admission, respectively). History of heart disease and chronic pulmonary disease remained significant factors that increased the risk of death (HR:1.74, 95%CI: 1.31-2.31; HR:1.34, 95%CI: 1.08-1.66). Diabetes, dementia, other unspecified comorbid conditions were also significantly associated with shorter time to death in the period one-to six-months (see table).

HR for time to death after six months were statistically significant only for age, male gender, dementia and comorbidities grouped as "other diseases". There was a significant protective effect of surgery performed after two days of admission and elapsed time after surgery > 2 days (see table for HR).

## Discussion

We evaluated survival rates in a cohort of elderly people, ≥ 65 years old, admitted in 2006 to hospitals in the Lazio region, Italy, after a hip fracture. Many studies have evaluated factors associated with survival after hip fracture but the majority have been interested in factors related to surgery [6,9,17,19,27] or in comparing mortality rates between different hospitals and different geographical areas [10,11,28]. We provided more specific analyses to evaluate factors associated with early (within one month), intermediate (1 to six months) and late (6 to 24 months) survival after admission. It is of note that only few studies have explored factors associated with short and long term survival [18,29].

This study confirms the high risk of mortality and of hospital re-admission shown in numerous others on hip fractures in the elderly [6,11]. The risk of death is very high in the first month (0.79 per person-year (PY)], and then declines thereafter (0.31, and 0,10 from 1 to six months and from 6 to 24 months, respectively). Overall, mortality at two years after index admission was 26% and was significantly higher among men (37% vs. 22% of women) (p < 0.05). Twenty percent had at least one more acute care hospitalization, 16% of patients did not have the surgery and only half of the cohort (49.4%) received rehabilitative care. Similar to the findings of other surveys, both national and international [1,17,28], in-hospital mortality during the first hospital admission was 5%, accounting for less than 20% of the total deaths.

From the early mortality model, there was a risk associated with advanced age, male gender, to being unmarried and to the presence of certain diseases such as cardiovascular, pulmonary and renal disease. For intermediate term mortality, male gender and age remained significant with higher HRs compared to those from early mortality. Moreover, in the intermediate term we observed a decrease in risk for cardiovascular disease, and a significant association for diabetes and dementia that in early mortality were not significant. Late term mortality remained significantly associated with age, male gender, cognitive problems and the presence of other comorbidities.

These results are consistent with the most recent literature. Previous work has shown that older age and male gender are significantly associated with mortality up to 5-10 years after the fracture [11,30] and cognitive problems are significantly associated with mortality at 12 and 24 months after sustaining a fracture with a 1.5 times higher mortality risk at one year than those without comorbidities upon admission [20,31]. British [19] and American [10] studies showed increased mortality with age, in presence of comorbidity and in males. Another Canadian cohort study reported that advanced age and 10 different comorbidities were independently associated with mortality [18]. Paksima and colleagues [11] found that patients with pulmonary or cardiovascular disease had a 27% and 40% higher risk of death compared to those without comorbidity. Additionally, other studies in accordance with the risk of early mortality observed in our study for widowed or divorced patients reported that poor health, and lack of family and social relationships are negative prognostic factors, in terms of both mortality and morbidity [32].

Timely enrolment in a rehabilitation program can notably reduce adverse outcomes resulting from a hip fracture, can support the recovery of mobility and daily activities, as well as significantly reduce the incidence of repeated hospital admissions [5]. In accordance with the literature, we found a risk reduction when accessing to rehabilitation care both in the early and the intermediate mortality model whereas the association was no longer significant in late mortality. The observed reduction in mortality for those who have access to rehabilitation indicates the need for ad hoc evaluations, which thoroughly investigate the aspects that cause local and organizational barriers to rehabilitation [33]. On the contrary we cannot exclude that there could be a selection bias, even controlling for the other factors, with patients in better conditions that start rehabilitation care.

Many studies indicate high rates of mortality for nonsurgical patients, especially after one year. Surgical treatment compared with conservative treatment, in fact, reduces the likelihood of leg deformities, reduces the length of admission, and leads to a more effective rehabilitation [3-34]. Not everyone, however, agrees on a relationship between preoperative hospitalization and mortality [9,16,17]. In our study, postoperative mortality was 19.7% at two years. We performed models that permitted to distinguish a significant increased of risk in the first two day after surgery and a significant reduction thereafter with this effect still evident also for late mortality. The increased risk of death in the first two days after surgery is probably due possible postoperative complications, as well as to those factors, not identified in this study, such as organization and preoperative evaluation. Roche and colleagues [19], in a prospective study on post-operative complications among the elderly operated on for hip fracture, reported that among patients with multiple comorbidities, there is a high risk of developing pulmonary and cardiac complications following surgery associated with 3 and 8% higher 30-day mortality, respectively, than those without comorbidities.

Assessing the risk of mortality linked to annual surgical volume of a facility is more controversial: this work showed a significant increased risk in the period 30-180 days after hospital admission for those who had been recovered in hospitals with a medium volume of hip fracture surgery. This result is similar to that of another Italian study [17], yet there are other recent studies on the subject that have not revealed any differences [35]. Contrasting results could be due to the fact that volume activity is an indicator of other characteristics of the facility that are not always easy to assess or residual confounding due to a worse case-mix in larger hospitals.

The multilevel analysis introduced in the Cox model (shared frailties Cox regression model), showed that there is a correlation between patients recovered in the same hospital (theta < 0.05), therefore we have a heterogeneity between the hospital with a latent common group effect. The model used assumes, in fact, that survival times can be considered independent, conditionally on the random effect shared. The statistical model used took into account the non-independence of observations at the same time allowing for the simultaneous analysis of the variables that "come" from the different facilities.

The study has some limitations that need to be highlighted. First, the cohort was analyzed only in relation to the explanatory variables from the index hospitalization and with little additional information related to medical conditions and complications after discharge. In addition, health databases were used that were not created exclusively for epidemiological analyses.

The limits described above, however, can be counterbalanced with some merits. The study is "area-based" and no selection was made of patients or of medical facility - it is perfectly representative of the real circumstances of an Italian region. Furthermore, an original feature of the study is that we investigated the determinants of early, intermediate and late mortality from hospital admission by integrating various medical archives, (i.e., mortality registry, RHD, and the Inpatient Rehabilitation registry) providing additional information useful to evaluate parameters potentially related to the outcome.

## Conclusions

Our study suggests that both clinical and organizational factors of acute care treatment are associated with the risk of early mortality and that risk increases when we consider intermediate term mortality. In two-year post-admission mortality, however, only age, male gender, cognitive problems and the presence of other comorbidities remain significant but with less risk than in earlier periods. This result could indicate that, among those who survived 6 months, other factors occurring after the fracture event play a important role.

## Competing interests

The authors declare that they have no competing interests.

## Authors' contributions

EC conceived of the initial study idea, performed the statistical analysis, drafted and revised the manuscript. PP helped to conceive of the study, supervised all phases of the study (i.e., data linkage, activities coordination, data analysis), drafted and revised the manuscript. AF helped to conceive of the study, supervised the initial phase of the study, revised the manuscript. DDL helped to conceive of the study, revised the manuscript and contributed especially to the intellectual content. GG supervised all phases of the study and revised the manuscript.

All authors read and approved the final manuscript.

## Pre-publication history

The pre-publication history for this paper can be accessed here:

http://www.biomedcentral.com/1471-2318/11/37/prepub
